# Models and impact of patient and public involvement in studies carried out by the Medical Research Council Clinical Trials Unit at University College London: findings from ten case studies

**DOI:** 10.1186/s13063-016-1488-9

**Published:** 2016-07-29

**Authors:** Annabelle South, Bec Hanley, Mitzy Gafos, Ben Cromarty, Richard Stephens, Kate Sturgeon, Karen Scott, William J. Cragg, Conor D. Tweed, Jacqueline Teera, Claire L. Vale

**Affiliations:** 1 Medical Research Council Clinical Trials Unit at UCL, Aviation House, 125 Kingsway, London, WC2B 6NH UK; 2 North Yorkshire AIDS Action, 20 St Saviourgate, York, YO1 8NN UK; 3 National Cancer Research Institute Consumer Forum, Angel Building, 407 St John Street, London, EC1V 4AD UK

**Keywords:** Patient and public involvement, Consumer involvement, Clinical trials, Systematic reviews, RCTs

## Abstract

**Background:**

Patient and public involvement (PPI) in studies carried out by the UK Medical Research Council Clinical Trials Unit (MRC CTU) at University College London varies by research type and setting. We developed a series of case studies of PPI to document and share good practice.

**Methods:**

We used purposive sampling to identify studies representing the scope of research at the MRC CTU and different approaches to PPI. We carried out semi-structured interviews with staff and patient representatives. Interview notes were analysed descriptively to categorise the main aims and motivations for involvement; activities undertaken; their impact on the studies and lessons learned.

**Results:**

We conducted 19 interviews about ten case studies, comprising one systematic review, one observational study and 8 randomised controlled trials in HIV and cancer. Studies were either open or completed, with start dates between 2003 and 2011. Interviews took place between March and November 2014 and were updated in summer 2015 where there had been significant developments in the study (i.e. if the study had presented results subsequent to the interview taking place). A wide range of PPI models, including representation on trial committees or management groups, community engagement, one-off task-focused activities, patient research partners and participant involvement had been used. Overall, interviewees felt that PPI had a positive impact, leading to improvements, for example in the research question; study design; communication with potential participants; study recruitment; confidence to carry out or complete a study; interpretation and communication of results; and influence on future research.

**Conclusions:**

A range of models of PPI can benefit clinical studies. Researchers should consider different approaches to PPI, based on the desired impact and the people they want to involve. Use of multiple models may increase the potential impacts of PPI in clinical research.

**Electronic supplementary material:**

The online version of this article (doi:10.1186/s13063-016-1488-9) contains supplementary material, which is available to authorized users.

## Background

Patient and public involvement (PPI) in research describes a variety of activities ensuring that research is carried out in collaboration with patients and/or members of the public [http://www.invo.org.uk/find-out-more/what-is-public-involvement-in-research/]. There are a number of arguments to support PPI. In publicly funded research in particular, these revolve around accountability and democracy [1]. On a more practical level, many researchers believe that involvement improves the overall quality of the study and increases the likelihood that a study will be successful [2–5]. Increasingly, many funding bodies are encouraging or insisting upon some form of PPI in the clinical studies they fund.

The MRC Clinical Trials Unit (MRC CTU) at University College London (UCL) designs, conducts, analyses and reports high-quality randomised controlled trials (RCTs), meta-analyses and other clinical studies in a variety of health-care areas, primarily cancer, HIV and other infectious diseases. The unit is committed to active PPI in all its clinical research and has developed a policy and accompanying guidance to support this (these documents, or details of how to obtain them, can be found at http://www.ctu.mrc.ac.uk/resources/patient_involvement). This work is overseen by a PPI steering group, which is currently made up of eight members of staff from each of the major areas and types of clinical research carried out by the unit (cancer, HIV treatment and prevention, tuberculosis, RCTs, meta-analysis and observational studies), two patient representatives (one involved in HIV research and one primarily in cancer) and two external PPI experts.

We previously reported the results of a survey exploring PPI in the unit, from the researchers’ perspective [6]. Researchers were asked to describe PPI activities in studies spanning more than 20 years. Whilst there was little or no involvement noted in the older studies, the majority of the more recent studies reported some PPI activities (e.g. patient representation on a formal trial group or committee). Encouragingly, most of the researchers who participated in the survey perceived benefits of PPI in their research.

The PPI steering group became aware that a variety of different approaches to PPI were being used in newer studies in the unit. These varied dependent upon study characteristics including disease, setting, population, geography, the stage of the study and the specific aims of the involvement. The group developed a series of case studies of PPI with the aim of documenting the models used and their aims, to gain a better understanding of PPI across different types of research being conducted within the unit. Furthermore, we aimed to highlight innovative PPI for future research and share examples of good practice.

Much of the literature of PPI in clinical trials is reports of single case studies [7] or surveys with, necessarily, less detail about the context of individual studies and PPI approaches used [8]. There have been several publications that have looked systematically at PPI in a cohort of trials covering a range of diseases [9–11]; however, these have been limited to trials taking place in a single country (the UK). Here we present the findings from case studies covering a range of geographical settings, study designs and patient populations, allowing us to make comparisons.

## Methods

The original idea for this work came from a participant at a workshop for involved patients and patient organisations hosted at the unit in June 2013 (workshop report available from: http://www.ctu.mrc.ac.uk/13706/13710/promoting_patient_and_public_involvement_in_clinical_trials_at_the_medical_research_council_clinical_trials_unit). We used purposive sampling to identify a cohort of studies representing the scope of our research and different approaches to PPI, and the geographical range of our studies. Our definition of PPI is based on that of INVOLVE (http://www.invo.org.uk/find-out-more/what-is-public-involvement-in-research/), and is the active involvement of patients, carers or family members, health service users, patient representatives or members of groups or organisations that represent those affected by the condition being researched as partners in the research process.

Once we had identified the cohort of studies, the lead researcher (or other key staff member) was invited to participate in semi-structured interviews around PPI within the study. We asked the researcher we interviewed from each study to nominate one or more patient, lay or community representatives who had been involved in the study to be interviewed also. None of the patient, lay or community representatives we interviewed were participants in our cohort of studies. We (BH) conducted these face to face wherever possible (otherwise over the telephone), following a topic guide designed to capture qualitative information on who was involved, the aims of involvement, the types of activities carried out by those involved, the support provided to those involved, the costs of PPI and its impact on the studies. It also included questions on motivation for involvement and lessons learned for the future. The full topic guide is provided in the Appendix.

Application of the National Health Service (NHS) research ethics committee Health Research Authority decision-making tool (http://www.hra-decisiontools.org.uk/ethics/) indicated that because our research was not a clinical trial of a medicinal product or device, no clinical data were collected and the interviewees were neither identified from or because of their past or present use of services, ethics approval was not necessary, and on this basis, was not sought. The UCL research ethics committee exempts ‘Research involving the use of non-sensitive, completely anonymous educational tests, survey and interview procedures when the participants are not defined as “vulnerable” and participation will not induce undue psychological stress or anxiety’ from the requirement to seek ethical approval from the committee. As our study met these criteria, as a service evaluation of PPI within our trials, ethical approval from the UCL research ethics committee was not sought. Interviewees gave verbal consent to being interviewed, and for the notes of these interviews to be analysed and published.

The face-to-face interviews took place in meeting rooms at the office of the unit. The interviews lasted approximately 45 min. The interviews were not audio-recorded and transcribed, as this project was initially designed to gather information for internal use rather than publication. Written summaries of each interview were produced instead, as a less time-consuming approach. Inaccuracies or inconsistencies were resolved with the interviewees, who also verified the final versions. One author (AS) then analysed the content of the summaries, following a process of familiarisation with the data, creating tables with data extracted from the written summaries organised by topic (based on the interview guide) to allow for comparison across the case studies, and identifying initial themes. The tables and initial themes were shared with the MRC CTU PPI steering group, who discussed and agreed the themes (BH, BC, RS, KSc, CV and AS). The group also discussed and agreed the final interpretation (BH, BC, RS, KSc, CV, AS, KSt, WJC, CDT, JT and MG). Responses from research staff and PPI contributors were considered separately where possible and appropriate.

## Results

The unit currently has around 50 active clinical studies (predominantly RCTs). We identified 11 studies (nine RCTs, one observational study and one meta-analysis) that we wanted to do case studies on to represent the diversity of types of clinical studies (design, geographical setting, disease and patient population) and approaches to PPI within the unit. We approached the lead researcher at the unit for each of these, of whom ten out of 11 agreed for a case study to be done on the PPI within their study. The reason given for not agreeing was that the researcher felt that there had not been much PPI in their study. We interviewed all patient representatives who the lead researcher identified and put us in touch with. We conducted 19 interviews with 13 researchers and seven patient representatives (one interview included two researchers from the same study), collecting information on eight individual RCTs [12–19] and one observational study [20], covering either cancer or HIV. The interviews took place between January and November 2014. In addition, data that had been collected for a previous project from two researchers and four patient representatives regarding PPI in a meta-analysis [21] were included for completeness, and provided the data for the analysis.

Most of the patient representatives interviewed were directly affected by the condition being studied in the trial, while one had been a carer for someone with the condition and two were employed by patient groups. The studies included six that were primarily carried out in the UK, two in sub-Saharan Africa and two across multiple continents. Table 1 gives an overview of the studies included and the full interview summaries are available upon request. The full interview notes can be found in Additional file 1.

**Table 1:** Overview of included studies, models and impacts of PPI

Study (and status at time of interview)	Disease	Patient population	Interviewees	Model of involvement	Patient or community representative	Area of impact reported (based on PiiAF)^a^
Cohort studies						
AALPHI (ongoing)^a^	HIV	Young people in the UK who are HIV-infected or live with someone who is HIV-infected	• Research nurses • Staff member from a voluntary organisation that supports young people living with HIV	• Patient representative on the steering committee • Representatives from voluntary organisations on steering committee • Patient groups facilitating involvement in specific activities	• Young people living with or affected by HIV • Patient groups or voluntary organisations that work with young people with HIV	• Research design and delivery • Ethics • Recruitment
Randomised controlled trials						
BREATHER (ongoing)	HIV	Young people with HIV living in Africa, South America, Asia, Europe or North America	• Researcher	• Patient groups facilitating involvement in specific activities • Adult representative from a patient group on the trial steering committee • Participant meetings in Uganda	• Children and young people linked to the Children’s HIV Association • Representative of patient group • Trial participants	• Agenda • Dissemination
DART (completed)	HIV	Adults living with HIV in Uganda or Zimbabwe	• Trial manager	• Community representatives on trial steering committee • Peer support groups for trial participants	• President of the Market Vendors’ Association in Kampala, Uganda, and partner institution’s community representative in Zimbabwe	• Recruitment • Dissemination
Microbicide Development Programme (MDP301) (completed)	HIV	HIV-negative women living in South Africa, Tanzania, Uganda or Zambia	• Researchers	• Community liaison officers • Community advisory boards • Community meetings • Site-specific approaches • Participant advisory groups	• Elected community representatives • Trial participants • Community members • Media • Other stakeholders	• Research design and delivery • Ethics • Dissemination
PIVOT (completed)	HIV	Adults living with HIV in the UK	• Researcher • Patient representative on the trial steering committee • Patient representative on the data monitoring committee	• Patient representative on trial steering committee • Patient representative on data monitoring committee	• UK community advisory board were asked to nominate members	• Agenda • Research design and delivery • Recruitment • Analysis of data (choice of comparator) • Dissemination
PROUD (ongoing)	HIV	HIV-negative men who have sex with men in the UK who are at high risk of HIV	• Researchers • Lay member (and co-chair) of the trial steering committee and community engagement group	• Community engagement group • Community representatives on trial steering committee (including joint chair) • Community representatives on trial management group • Participant meetings • Community representative on data monitoring committee	• Representatives from community groups • Trial participants	• Agenda • Research design and delivery • Ethics • Recruitment • Analysis of data (interpretation) • Writing-up • Dissemination
QUARTZ (ongoing)^a^	Non-small-cell lung cancer	Adults in the UK whose non-small-cell lung cancer has spread to their brain	• Researcher • Lay member of trial management group	• Patient representative on trial management group	• Lay member of the NCRI CLG and carer for someone with lung cancer	• Research design and delivery • Recruitment
SORCE (ongoing)^a^	Kidney cancer	Adults in the UK with kidney cancer	• Researcher • Patient representative on trial management group	• Patient representative on trial management group	• Lay member of NCRI renal cancer CSG and trustee at Kidney Cancer UK, with personal experience of the disease	• Ethics
STAMPEDE (ongoing)^a^	Prostate Cancer	Men with prostate cancer in the UK or Switzerland	• Researcher • Trial manager • Patient representative on trial management group	• Patient representatives on the trial management group	• Patient representatives with personal experience of the disease	• Ethics
Individual participant data meta-analysis						
Cervical cancer meta-analysis (completed)	Cervical cancer	Women with cervical cancer who took part in RCTs worldwide	• Researcher • Patient research partners	• Patient research partners	• Five patient representatives with personal experience of the disease	• Agenda • Analysis of data (interpretation) • Writing-up

^a^Analysis, writing-up and dissemination are not yet available for ongoing studies

The main approach to recruiting patient and community representatives was via patient groups or by approaching people who were already involved in other research (for example, through the National Cancer Research Institute (NCRI) Consumer Liaison Group (CLG)). Three studies [12, 13, 16] involved study participants (in addition to patient or community representatives who were not taking part in the study) directly and tended to do so through advertisements in clinics and/or the study website and participant mailing list. For most of the case studies, little specific information was reported about the additional costs associated with PPI, so we do not present those findings here.

We categorised the findings into aims and motivations for PPI, models of involvement, impacts of involvement and lessons learnt. These findings are presented below.

### Aims and motivation for PPI

We found that the trial teams broadly agreed on their reasons for involvement. All researchers interviewed said that PPI gave them the opportunity to understand better the perspectives of the affected population. Some of the researchers interviewed were more specific, saying that PPI had provided help with issues around recruitment and/or retention, and, in the case of the two HIV prevention trials, to ensure acceptability of the study. Two trials also mentioned that PPI was a condition of their Health Technology Assessment (HTA) funding.

There were also considerable similarities in the motivations patient representatives cited for being involved in the studies; for example, wanting to help with research that they felt was addressing an important question (8/11 patient representatives). While being affected by the condition (or caring for someone who was) was often a criterion for becoming a patient representative on these studies, it was not explicitly cited by the patient representatives as a motivating factor. Other reasons mentioned included interest in research, a desire to learn more about clinical trials, a desire to put forward a patient’s perspective, and a feeling that they had the time and skills needed to contribute.

### Models of involvement

In this section we describe the models of involvement in terms of who was involved in the PPI, the types of activities included and the support offered. Table 2 summarises the main models of involvement used in the study.

**Table 2:** Models of involvement used in our case studies

Role	Managerial	Oversight			Responsive					
Models	Patient/public representative on trial management group	Patient/public representative on trial steering committee	Patient/public representative on data monitoring committee	Patient research partners	Involvement on specific tasks facilitated through existing patient groups	Ad hoc participant meetings	Ongoing participant groups	Community advisory groups specific to study	Community advisory groups providing advice across several studies	Community meetings to advise trial teams
Who is involved?	Patient or public representatives					Study participants		Community members		
Duration of involvement	Long term				One-off	Ad hoc	Long term			Ad hoc
Studies using this model	PROUD QUARTZ SORCE STAMPEDE	AALPHI BREATHER DART PIVOT PROUD	PIVOT PROUD	Cervical cancer meta-analysis	AALPHI BREATHER	BREATHER PROUD	DART MDP301	MDP301 PROUD	MDP301	MDP301

The most common models of PPI were having patient or community representatives on either the trial steering committee (five studies) or the trial management group (three studies), reflecting the typical management structures of the cancer and HIV trials within the unit. HIV trials (AALPHI [20], BREATHER [17], DART [12], PIVOT [18] and PROUD [16]) commonly involved community members or patients on the study’s steering committee, a high-level group that oversees the conduct of the study. For the cancer trials (QUARTZ [14], SORCE [19] and STAMPEDE [15]), involvement was on the trial management groups, which are responsible for the set-up and day-to-day running of the trial and release of results or publications. Several patient or community representatives who were involved in these types of formal trial committees were people who already had experience of PPI activities, or were active in patient groups or organisations. They tended to have a good understanding of research processes and/or the disease area and had established links with the patient community. Two HIV studies had patient or community representatives on the data monitoring committee. Where studies involved patients or community representatives on formal committees, they tended to involve one or two representatives per committee (although the individuals may change over the course of the study). Two studies (QUARTZ and SORCE) involved only one representative in total. In general, meetings between researchers and patient or community representatives were held either face to face or by telephone. Payment and provision of support for patient and community representatives were mixed such that for some studies, there was no budget to enable payments to be made, but for the cancer trials (QUARTZ [14], SORCE [19] and STAMPEDE [15]) and the AALPHI study [20], the representatives’ expenses were covered and a fee was paid. Although most of the patient and community representatives received no formal training, most said that they were offered background information at the start of the study and also talked about how MRC CTU staff and/or other committee members had been helpful when they had questions.

In addition to representation on trial oversight or management groups, the HIV studies each employed other models of involvement. For example, patients and community members were involved in peer support groups, participant meetings or community engagement groups, some of which had formal membership and regular meetings, while others were ad hoc. We do not have data on how many patient and community representatives were involved in these more responsive roles for each of the case studies that used these approaches, although it was likely to be 5–20 per task for AALPHI and BREATHER, with many more than that involved over the course of MDP301 and PROUD. They involved a broad spectrum of people, many of whom would have had no previous involvement or experience. Some studies worked with community organisations to facilitate involvement, in particular, the studies involving young people affected by HIV. For example, in the AALPHI study [20], community groups worked with young people to test interviews, improve the design of study materials and develop a video explaining the study to potential participants. In the BREATHER trial [17], the Children’s HIV Association facilitated a meeting with researchers, children and young people to discuss the study and to define topics for the qualitative interviews.

The single meta-analysis [22] involved five women, each of whom had experienced cervical cancer, as a group of research partners. Their role included helping to trace contact details for trial investigators, contributing to project newsletters, helping with the organisation of a collaborators’ meeting, providing input to the lay summary and a key messages document, and taking part in discussions about future research.

### Impacts of PPI

All interviewees felt that PPI had impacted on the study, although the type of influence varied. Overall, the impacts were categorised into seven of the nine key areas of impact outlined in the public involvement impact assessment framework (PiiAF, http://piiaf.org.uk/). In our case studies, we had no examples of PPI impact on data collection or time and cost. Table 1 includes the areas of impact reported in each of the case studies.

#### Influence on research design and delivery

Six of the studies reported that PPI had a positive influence on the research question and/or design of the study. For example, in the QUARTZ trial [14], researchers anticipated that there may have been problems collecting quality of life data from patients, as they had very advanced stages of lung cancer and may have been too unwell to respond. However, in response to a suggestion from the PPI representative on the trial management group, the final study design included collection of responses from carers, as a proxy for the patients. In the MDP301 trial [13], potential participants voiced their concerns about the ability of clinics to sterilise the instruments being used in the clinics at community engagement meetings, resulting in the use of disposable instruments in the trial. In the PROUD study [16], the community engagement group had concerns that the use of a placebo control might hamper recruitment. They advocated instead for the use of a deferred control arm and also for a simple, daily dosing schedule, thus directly influencing the design of the study. Finally, a patient representative on the PIVOT trial steering committee raised concerns that recruitment to a sub-study of the trial may be problematic, as the study involved patients having a lumbar puncture. As a result, the sub-study was redesigned and the target number of participants was reduced substantially.

#### Impact on ethics and recruitment

The PiiAF framework classes are ‘improving the consent process by producing clearer more accessible information’ under the heading of ‘ethics’. We believe that improvements in communication with potential participants can have (and in some of our studies, have had) an impact on recruitment to the study. Therefore, we look at these two areas of the PiiAF framework together. Eight studies reported improved communication with potential participants and/or impacts on recruitment as a benefit of PPI. For example, the patient representative on the QUARTZ trial management group gave talks about the trial at meetings that led to new clinicians signing up to the trial. Patient representatives on the SORCE and STAMPEDE trial management groups identified the need for clearer and simpler patient information sheets. Young people worked with a community organisation to design a logo and posters to promote the AALPHI study [20] and produced a short video to explain the study in a way that is fun and understandable.

In MDP301 [13], regular meetings with participants through the duration of the trial informed recruitment and retention and communications and dissemination strategies. They also helped to hone the trial messages. In addition, the community advisory board and participant stakeholder group worked with the research team to provide a swift and appropriate response to community concerns about the closure of another microbicide study. This helped to avoid any potential negative impact on the MDP301 study. Researchers on the DART trial [12] also felt that community involvement was a key contributing factor to the very high levels of recruitment and retention in the trial, through creation of a sense of a DART community.

HIV trials in the UK often struggle to recruit participants of African origin. The PIVOT team [18] worked with the African Eye Trust to address this. Trust members gave presentations about the trial at a number of hospitals and clinics, and included an article about the trial in their magazine. More than 20 % of PIVOT participants were of African origin, which is a much higher proportion than for many other UK HIV studies.

#### Giving researchers the confidence to do or continue a study

Trials do not always go smoothly; for example, recruitment may be slower than anticipated and researchers then have to make the difficult decision on whether to continue the trial. In the case studies, researchers, including from the QUARTZ [14] and PROUD trials [16], reported that PPI had given them the confidence to keep going with a study when recruitment was slower than anticipated. The researchers also reported that patient or community representatives had reassured them of the importance of the trial.

Researchers on the STAMPEDE trial [15] highlighted the important role that two patient representatives on the trial management group had played prior to the trial opening. A drug similar to one of those due to be investigated in STAMPEDE was withdrawn from the market due to safety concerns and all trials involving this family of drugs were stopped indefinitely. The STAMPEDE trial management group had to decide whether to drop this drug from the trial or to wait until there was further guidance from the regulatory authorities. The patient representatives played a key role in assessing the potential risks and benefits of the drug for men with prostate cancer, and in the decision to wait rather than going ahead without it.

In PIVOT [18], the patient representative on the data monitoring committee persuaded the committee to continue with the study at least until further evidence was available, following a serious adverse event to a participant that may or may not have been related to the trial treatment. Having the view of a patient on the balance between risk and benefit gave the members of the data monitoring committee more confidence in their decision.

#### Impact on analysis, writing-up and dissemination

There was considerable overlap in what interviewees reported in terms of the impact on analysis, writing-up and dissemination, therefore they are discussed together. Three studies reported that PPI had impacted on the interpretation and communication of the results. For example, when preliminary results of a PIVOT sub-study on cognitive impairment were presented at an investigators’ meeting, the patient representative on the trial steering committee was concerned about the language used and the appropriateness of the analysis, which had not taken socio-economic and cultural factors into account. This led the researchers to make appropriate adjustments to the analysis before the results were more widely disseminated. Similarly, the researchers involved in the PROUD trial [16] held a number of meetings to gather feedback from participants on the study results, which led them to change the emphasis of the key messages prior to their public release. Initially researchers had planned to emphasise in the key messages the high risk of HIV faced by this group, but following feedback from participants, the emphasis was reduced due to a concern about stigmatisation of the participants.

Three of the patient research partners on the cervical cancer meta-analysis [22] wrote a joint article with the researchers to describe the side effects of cervical cancer treatment from a patient’s perspective [23], helping to place the results in the context of patient experience.

#### Impact on future research agenda

The case studies also highlighted that where there is PPI in an ongoing or ending study, there is an opportunity for PPI input into future studies, which often follow on from existing studies, and may involve many of the same researchers and patient representatives. For example, the patient research partners on the cervical cancer meta-analysis raised concerns about the lack of information on the late side effects of cervical cancer treatment available from the meta-analysis. This led to researchers collaborating in a separate research project with a greater focus on late side effects.

Another example is that the patient representative on the SORCE trial management group became involved in the trial development group, designing a follow-up study to SORCE on renal cancer.

As recruitment to the PROUD pilot study came to an end, participants contributed to discussions about the future research priorities for HIV prevention among men who have sex with men in the UK through participant involvement meetings via a webinar and teleconference. These were advertised through the study website, study newsletter and in study clinics. Their discussions helped to directly inform the development of a funding application to the HTA programme.

### Lessons learnt

Researchers and patient representatives were asked what advice they would give to researchers looking to involve patients and the public, and shared their key lessons for future studies. These can be broadly grouped into three areas: who to involve, types of involvement and providing support. Advice on who to involve centred on the skills, characteristics or background required and on recruitment. Lessons around types of involvement tended to be reflections of what worked well in the study the interviewee was involved in. Discussion of support included the support that can be given by the research team, support available from existing community or patient groups, and the support other patient representatives can provide. Further details are presented in Box 1.

## Discussion

In our study, the case studies demonstrate that a range of models and approaches to PPI have led to numerous benefits across the key areas of impact, with examples from the design phase through to interpreting and communicating the results of studies. Motivations for involvement, both from the researchers and from the patient and public representatives who were interviewed were broadly similar with an overall aim of improving the study.

These case studies follow on from a survey of PPI in MRC CTU studies, the results of which we reported previously [6]. Clearly, they do not provide a complete picture of all PPI in research carried out by the MRC CTU, or indeed, more widely. Our aim for this study was to provide a detailed look at the ten studies chosen, to provide clear illustrations and examples of PPI across the range of research activities in the MRC CTU and to evidence the impact of PPI on those studies. Although there are numerous difficulties in assessing the impact of PPI in clinical research, we found a variety of impacts that fit well into the framework of public involvement impacts on research identified by the PiiAF project (http://piiaf.org.uk/). These covered each of the areas of agenda, research design and delivery, ethics, recruitment, analysis of data, writing-up and dissemination. Like the case studies evaluated by Evans et al. [24, 25], PPI was reported to have had some impact in all our studies, particularly in recruitment and materials. Like the EPIC survey of HTA-funded trials [25], none of our informants reported PPI as having had a negative impact on the study. It should be noted that a number of the case studies were about research that was still ongoing at the time of the interviews. Therefore, there are fewer examples of impact on the latter stages of the research process (analysis of data, writing-up and dissemination).

There is a growing body of literature around PPI; however, examination of the INVOLVE Evidence Bibliography reveals that most references that focus on PPI in clinical trials are case studies of individual trials [7], limiting generalisability. Our study adds to the existing literature through providing details of, and comparing and contrasting PPI undertaken in eight RCTs, encompassing different disease areas, geographical locations and approaches to PPI. The multiple case studies approach allows us to provide more depth than higher-level surveys across many trials, but as the sample was purposively selected, it lacks the generalisability of more systematic approaches, such as the EPIC study [9, 10].

Our analysis of the data is limited by the lack of audio-recording and verbatim transcriptions of the interviews. Relying on written summaries of the interviews may mean that we have missed key information. However, we are confident that the written summaries are accurate and contain the points considered important by interviewees, as all our interviewees were asked to read and correct or approve the written summaries prior to the start of analysis.

Our findings have enabled us to describe better the wide variety of approaches being used to involve patients and the public in our research. In keeping with the findings of a previous survey of how consumers were involved in NHS research [8], most often patient or community representatives were involved in trial steering committees, trial management groups and data monitoring committees. A systematic cohort investigation of HTA-funded trials also found that the most common approach to PPI was in a managerial role [10]. The representatives tended to be people who were already active in patient groups or other PPI activities. This has several advantages, including knowledge of research and the disease and having links with other patients. Having less formal approaches to PPI may offer different advantages. For example, it may help to involve people who are not comfortable in formal meetings and those who are unable to make a long-term commitment, which may particularly affect those from seldom heard groups. Although less likely to provide continuous long-term input, involving community or participant groups and close collaboration with voluntary organisations may offer better opportunities for more diverse voices to contribute to the research process. These approaches were predominantly used in the HIV studies, which tended to involve people in a wider variety of ways.

Perhaps the most novel finding of this study is the use of participant involvement by three of the HIV trials (including trials on prevention and treatment, and based in the UK and Africa). This is distinct from usual PPI practice in clinical trials, where patient or community representatives who are not taking part in the trial are chosen for involvement roles. This approach is recommended as Good Participatory Practice in biomedical HIV prevention trials [26], but we are unaware of it being reported in the formal literature elsewhere. None of the three studies used participant involvement as their only form of PPI, with researchers seeing it as complementing their other PPI approaches to answer questions that people who are not taking part in the trial cannot answer. We see no obvious reasons why participant involvement should not work equally well in other health-care research areas and are currently exploring how we might adopt them more widely at the MRC CTU at UCL. Participant involvement may have particular advantages where the intervention being tested is particularly novel (like MDP301 and PROUD), so that other patients or community members are unlikely to have experience of the issues that come with the approach. It may also be advantageous in disease areas where there are not already strong, established patient groups from which to recruit patient representatives.

There were no direct contradictions between the accounts of researchers vs patient representatives. There were some examples of an impact that were cited by one interviewee and not the others for a single study, perhaps because the other person interviewed may have been unaware of it. For example, the QUARTZ researcher said that PPI had given them the confidence to keep going with a trial that was struggling to recruit. The patient representatives did not mention this as an example of an impact, and subsequently told us he had been unaware of it until we fed the results of the case studies back to him.

Most of the literature on PPI to date has focused on studies in high-income countries (particularly the UK). Our case studies include three trials where all (DART and MDP301) or a large proportion (BREATHER) of participants are from low-income countries (particularly sub-Saharan African countries). The PPI models used in these case studies all include community representation on the steering committee (as did the other HIV case studies), and participant meetings, with ongoing participant groups for DART and MDP301. Only one of our studies based in the UK (the PROUD HIV prevention study) involved participants. Although there were similarities in the models of PPI used in the three studies taking place in Africa, there does not seem to be an obvious pattern in the areas of impact described by interviewees, suggesting that the area of impact is not solely related to the model of involvement.

Half the examined studies combined a mix of different PPI models, from long-term involvement in a formal steering committee through to one-off work to produce a video to promote the study. For example, in the PROUD study, there was long-term involvement of community representatives in the trial management group, trial steering committee and data monitoring committee, and in the community engagement group. There was also one-off involvement of participants in meetings to discuss specific questions (e.g. how to improve recruitment, what the next priority research question was, and how to interpret and communicate the results). In the EPIC study, multiple approaches within a study were only identified in six out of 89 outline applications for HTA funding, so it seems that this is not widespread [10]. In our study, the use of a variety of different approaches to PPI within a single study was seen only in the HIV studies and not in the cancer studies. The reasons for this are not clear, but may in part be due to the different cultures and traditions of PPI in these two disease areas, and to the experience of the senior researchers and teams involved in the studies. Where studies used a mix of models of PPI, it is not possible to attribute specific impacts to specific models. However, we feel that certain models may be more or less likely to influence particular aspects of the research. For example, the involvement of experienced patient representatives on formal trial groups or committees may be more likely to influence how studies are analysed than the one-off involvement of participants who may have less knowledge of clinical trials and who are less familiar with the process and terminology. However, the one-off involvement of trial participants may be best placed to react to and resolve a specific problem that arises during a trial (e.g. recruitment issues). Indeed, in studies of children or young people, or very mobile populations, who may be less able to contribute to ongoing formal committee structures, short-term, more informal models may be the only realistic option for active PPI. Although this is likely to have resource implications, we feel that combining different approaches may be cost-effective in the long term by maximising the potential impacts of PPI on a study.

We recognise that different approaches to PPI will have been adopted in other health-care areas and at other clinical trials units. Broader sharing of these experiences, their benefits and challenges may further benefit research practice.

## Conclusions

Our findings demonstrate that even within a single trials unit, a range of models of PPI are being employed to benefit clinical studies. Use of multiple models, possibly at different stages of the research process, may maximise the potential impacts of PPI in clinical research. Sharing experiences of the different approaches being used, across disease conditions and between clinical trials units, as well as an exploration of the impact and benefits of these approaches, is desirable. We recommend that researchers familiarise themselves with a range of methods. In partnership with patient representatives, they should consider which method(s) might be most appropriate, based on the desired impact, the people they want to involve and the time and resources available to support PPI.

## Abbreviations

AALPHI, adolescents and adults living with perinatal HIV cohort (AALPHI is a cohort study of young people infected with HIV at birth and a HIV-negative comparison group); BREATHER, a trial of short-cycle therapy (5 days on/2 days off) in young people with chronic HIV infection; CLG, consumer liaison group; CSG, Clinical Studies Group; DART, Development of Antiretroviral Therapy in Africa (a randomised trial of monitoring practice and structured treatment interruptions in the management of antiretroviral therapy in adults with HIV infection in Africa); HTA, Health Technology Assessment (a National Institute of Health Research funding programme); MDP301, a phase III trial to test the effectiveness of PRO2000/5 vaginal microbicide to prevent HIV infection; MRC CTU, Medical Research Council Clinical Trials Unit; NCIN, National Cancer Intelligence Network; NCRI, National Cancer Research Institute; NHS, National Health Service; PiiAF, public involvement impact assessment framework; PIVOT, protease inhibitor monotherapy versus ongoing triple-therapy in the long-term management of HIV infection; PPI, patient and public involvement; PROUD, pre-exposure option for reducing HIV in the UK (an open-label randomisation to immediate or deferred daily Truvada for HIV negative gay men); QUARTZ, quality of life after treatment of brain metastases (a trial looking at whether radiotherapy improves patients’ quality of life when lung cancer has spread to the brain); RCT, randomised controlled trial; SORCE, a multi-centre phase III double-blind placebo-controlled study designed to examine the efficacy and tolerability of sorafenib (Nexavar) in patients with resected (total or partial) primary renal cell carcinoma at high or intermediate risk of relapse; STAMPEDE, systemic therapy in advancing or metastatic prostate cancer (evaluation of drug efficacy); UCL, University College London; UK, United Kingdom

## Additional file

Additional file 1: Interview notes. This file contains notes from the interviews conducted with both researchers and patient representatives for the case studies. (PDF 423 kb)

## Appendix: Interview topic guide

### General

What was the study about? (One or two sentences in plain English)

Type of study (e.g. randomised, non-randomised, epidemiology, meta-analysis)

### For researchers

1. What was the aim of the involvement? What did you hope to achieve by it?
2. Who did you involve (e.g. members of a patient organisation or community group, members of the public)? Why did you choose these people?
3. How did you find people to involve? Did you have any problems finding people?
4. What methods did you use to involve people (e.g. membership of a trial management group, trials steering group, community advisory group)?
5. When were people involved (e.g. at what stages of the research process)?
6. What activities did people undertake (e.g. reviewing patient information sheets)?
7. How did you support people who were involved?
8. What did the involvement cost (time and money)?
9. What impact, if any, did the involvement have – on the research, on the researchers and/or on the patients/members of the public who got involved?
10. Any advice to other researchers thinking about PPI?

### For people who get involved

1. Why did you agree to get involved in this project?
2. What did you hope to achieve? And did you achieve it?
3. How were you involved?
4. Did you receive any support or training? If so, what was this and was it helpful?
5. Do you feel your involvement had an impact – on the project, on the researchers and/or on you?
6. Any advice to other researchers thinking about PPI?
7. Any advice to people who are asked to get involved in research?
