# Erratum to: ‘Models and impact of patient and public involvement in studies carried out by the Medical Research Council Clinical Trials Unit at University College London: findings from ten case studies’

**DOI:** 10.1186/s13063-016-1567-y

**Published:** 2016-09-07

**Authors:** Annabelle South, Bec Hanley, Mitzy Gafos, Ben Cromarty, Richard Stephens, Kate Sturgeon, Karen Scott, William J. Cragg, Conor D. Tweed, Jacqueline Teera, Claire L. Vale

**Affiliations:** 1 Medical Research Council Clinical Trials Unit at UCL, Aviation House, 125 Kingsway, London, WC2B 6NH UK; 2 North Yorkshire AIDS Action, 20 St Saviourgate, York, YO1 8NN UK; 3 National Cancer Research Institute Consumer Forum, Angel Building, 407 St John Street, London, EC1V 4AD UK

## Erratum

Unfortunately, the original version of this article [1] contained an error. Box 1 was omitted from the HTML version however the PDF version is fine and Box 1 can be seen there. To correct this we have included Box 1 below and will also be doing an update.

**Box 1 Lessons learnt: the views of researchers and patient representatives**

**Table:** 

**Who to involve:**
● Enthusiasm for research was considered an essential characteristic for patient representatives. There was less agreement over what other skills or attributes were essential. ● Patient representatives who had scientific backgrounds (in a variety of different areas of science) felt that helped them to adapt to the language and processes of medical research ● Trial participants can provide unique insights because they are going through the trial. This may be very valuable. ● Some patient and community organisations can help to match interested people with research with researchers, based on their relevant skills and experience and the focus of the research.
**Providing support:**
● Examples of support provided by researchers, that PPI representatives found to be useful include (but are not limited to): ○ Taking time to explain things to patient representatives ○ Helping patient representatives to prepare for meetings ○ Organising visits to trial sites to see how the trial runs ○ Providing a glossary of terms (including study-specific ones) that may be used. ○ Remembering that patient representatives may be personally affected by discussions. ○ Helping PPI representatives develop a better understanding of statistical concepts, especially at the analysis stage. ● Existing patient groups and community organisations can act as a vital bridge between researchers and patient and/or community representatives ● Building on existing community structures (such as community groups, work places or traditional leadership groups) rather than trying to create new ones can help to encourage community engagement (see MDP interview notes for more details) ● Having more than one PPI representative on a group or committee is important because: ○ They can help to support each other ○ It can help with the continuity of involvement, in particular where the study takes place over a prolonged period of time, or where there may be high turnover of patient representatives ○ It can help with diversity of involvement, in particular where there is considerable diversity in the population affected.
**Types of involvement**
● Integrating PPI at a cross-study or Unit level (e.g. Protocol Review Committee, or a panel of patient/community representatives to advise on several studies in a disease area) may facilitate PPI in the development and early phases of a study. ● It can be helpful to involve people who are already well known and have strong links to, patient groups/the community, particularly for involvement in more formal trial committees. ● Involving patients on data monitoring committees can be helpful, especially where side effects are an issue, as they can give a patient’s perspective on the balance between risks and benefits. ● Using a mix of PPI models, i.e. having different types of involvement at different stages of a trial and with different groups and individuals, may enhance the potential impacts of PPI.

## References

[1] South A, Hanley B, Gafos M, Cromarty B, Stephens R, Sturgeon K, Scott K, Cragg WJ, Tweed CD, Teera J, Vale CL (2016). Models and impact of patient and public involvement in studies carried out by the Medical Research Council Clinical Trials Unit at University College London: findings from ten case studies. Trials.

