# Supplementary material for: Models and impact of patient and public involvement in studies carried out by the Medical Research Council Clinical Trials Unit at University College London: findings from ten case studies
Source: Trials. 2016 Jul 29;17:376. doi: 10.1186/s13063-016-1488-9 (PMC4966697; doi:10.1186/s13063-016-1488-9)
Supplement: Additional file 1: — Interview notes. This file contains notes from the interviews conducted with both researchers and patient representatives for the case studies. (PDF 423 kb) [file 13063_2016_1488_MOESM1_ESM.pdf]

MRC CTU at UCL: PPI case studies

AALPHI: a cohort study of young people affected by HIV

What's the aim of the involvement?

The AALPHI study is looking at two groups of young people: perinatally HIV-infected individuals, who have been followed in childhood through the CHIPS study; and HIV negative individuals, who have a parent with HIV or who are living in the same household as an HIV-infected young person in the study, or have a sibling, friend or partner who is an HIV-infected young person in the study. Participants have 3 interviews over a 5 year period.

Researchers working on this study knew they would need to actively involve voluntary organisations working with young people infected and affected by HIV, and with the young people themselves, if they were to successfully recruit and retain participants.

Who is involved?

Voluntary organisations

The researchers have worked with voluntary organisations that support young people with HIV from the outset. The Projects Manager at CHIVA, the Children's HIV Association, has been key to the in the involvement of young people in the study acting as an advocate and advisor. She also gave feedback on the draft protocol and the participant information sheets.

A number of voluntary organisations, such as Body and Soul, Positive Parenting and Children and Positively UK are acting as recruiting centres for the study.

Young people

Young people were involved in AALPHI from the outset by piloting the interview. The first group of young people were from a local school in London, and then an updated version was tested by a group of young people from Body and Soul. Both groups commented on the participant information sheets as well. In addition a group of HIV positive young people from Bristol helped to design the study materials. They re-branded it as 'ALFIE' and designed a logo and a poster to help to recruit participants. Young people have also been involved in developing promotional material to try and engage negative young people in the study. They helped make a sock puppet video which explains what is involved if you join AALPHI. It was thought that presenting the study in a fun way with sock puppets might encourage negative young people, who are not used to going to being part of research studies, to participate. It is mainly being used in the voluntary sector in support groups for affected young people.

How are people involved?

The steering committee includes representatives from all of the large voluntary organisations that are involved in the study. The researchers spent some time thinking about how to involve young people who are affected by HIV on the steering committee. A young person, who was selected by a participating voluntary organisation, attended two meetings. They came with a supporter, and met with the 2 steering committee chairs thirty minutes before the start of the meeting so that they could discuss how they wanted to contribute. A new young person is now involved. She is a psychology student, so she has an academic interest in the study as well as a personal interest.

In future studies it is planned that researchers will involve a group of 4 or 5 young people, who will be involved in the design, pilots and the steering committee meetings. This will enable young people to build up a greater understanding of the study and provide some consistency while allowing for some change in the core group as well.

The researchers have set up a secret Facebook group for HIV-infected participants in the study. The plan is for the group to engage participants (i.e. keeping them up-to-date with the study), and to

use it to actively involve participants in the future by seeking their views on particular aspects of the study. If the group takes off it is hoped that the young person who sits on the project steering committee will be involved in running this group. However, to-date none of the 20 young people who have been invited to join the group so far have accepted to join, therefore other forms of social media, such as a whatsapp group or twitter may need to be explored in the future to see if they generate a better response.

The costs of PPI (both financial and time)

It is not possible to estimate the amount of time it has taken to actively involve voluntary organisations in this study as they are so integral to it.

Young people who took part in the pilot interviews were paid £30 each for their time, in common with all participants. If patients attend specifically for the AALPHI interview their travel is reimbursed. The young person who sits on the steering committee is also paid travel expenses and a fee.

What impact has the involvement had so far?

This study would not have been possible without PPI. When conducting a study using interviews, acceptability is a key issue. Young people have advised on what questions would be acceptable in the interviews, as well as on what clinical tests would be acceptable.

Voluntary organisations are acting as recruiting centres and have helped to promote recruitment. For example, one voluntary organisation that is also a recruiting centre for the study asked staff and volunteers to encourage their children to take part in the study. This meant that they were able to tell other parents what it was like to participate.

"PPI has to start at the design stage, as the study has to be acceptable. If it isn't, the participants either won't attend in the first place or won't come back a year later.

Young people are a transient group, so involving a group rather than one or two individuals is good."
Senior Research Nurses

The perspective of a community representative about involvement in the AALPHI study

The interviewee works with CHIVA, the Children's HIV Association.

Why did you agree to get involved in this study?

Because it's such an important study. I have contact with hundreds of HIV+ young people through my work at CHIVA, and they communicate really openly and honestly about their experience of HIV. We need to know what will happen to these young people as they grow up.

There is a lot of stigma attached to HIV. I was very interested in the neuro-cognitive aspect of the AALPHI study. It's important we understand what impact HIV has on the brain, but also what impact the stigma around HIV has on young people.

The paediatric HIV world is a small one – Ali Judd, the chief investigator, used to be a Trustee for CHIVA, and Marthe Le Provost, one of the researchers, used to work for CHIVA. I have been involved with paediatric HIV research for a number of years.

What did you hope to achieve?

I wanted to ensure that the interactions between the researchers and the young people were as good as they could be, and that the study used the best models of involvement.

We are still on a learning curve. Our learning on AALPHI and other studies has led to our plans for the involvement of young people in the proposed PENTA 20 trial, where we hope to involve a group of HIV+ young people from an early stage, and promote peer support and mentoring.

Do you feel your involvement had an impact?

I can see that we have had an impact on the people working on the study.

Because we spend a lot of time with young people and we have a youth committee to advise us, we were able to act as a bridge between young people and the researchers. We helped the researchers think about how best to communicate with young people who might be willing to take part in the study. Communication about research doesn't have to be dry – for example we developed a film about the AALPHI study in partnership with young people, using sock puppets.

Any advice to other researchers thinking about PPI?

If you are planning to involve young people, do it from the start of the research – it will set the ethos of the whole study. Do this in a non-tokenistic way, on their terms, using the right language. Find the organisations or individuals that act as bridges to the young people you want to involve – in the case of paediatric HIV this is CHIVA – and seek guidance from them.

Make sure that organisations that work with young people are aware of the research that is relevant to them. And that they can get actively involved.

It's better to involve a group of young people rather than one young person. Ensure you offer support and training and that you prepare them for any meetings. Make sure the young people you involve feel empowered to challenge what's going on.

BREATHER: A study exploring whether HIV affected children and young people can take weekends off from their treatment

The BREATHER study compared two different ways for children and young people with HIV to take anti-HIV medicines:

1. Taking the medicines every day (as most children in the UK and many other countries do now)
2. Taking the medicines during the week for 5 days and then having a break for 2 days (i.e. no antiretroviral medicines) at the weekend.

The study also asked children and young people for their views about these different ways of taking anti-HIV medicines.

What was the aim of the involvement?

There were a number of reasons for involving children, young people, their families and HIV community organisations in this study:

- There is a long history of partnership working in HIV research, so all HIV studies based at MRC CTU has some type of involvement.
- The subject matter of the BREATHER study meant that it was important to seek the views of children and young people from an early stage – a strong message to HIV affected people has always been how important it is to take medication every day, and this study explored whether this is always appropriate.
- The UK part of the study was funded by the National Institute for Health Research Health Technology Assessment Programme, which requires patient and public involvement as a condition of funding.

Who was involved?

In the UK, children, young people and staff from CHIVA, the Children's HIV Association, and a representative from i-Base, a treatment activist group that is committed to providing timely and up to date information about HIV treatment to HIV positive people and to health care professionals.

In Uganda, children and young people who took part in the study.

How were people involved?

At an early stage, researchers met with a group of children and young people from CHIVA to talk about plans for the study as a whole, and later to talk about the topic guides for the interviews.

A representative from i-Base sits on the BREATHER Trial Steering Committee.

In Uganda, researchers held study meetings for children and young people who took part in the study to keep them up-to-date with progress and to seek their views about the study. They also talked with children and young people informally and on a one-to-one basis.

At the dissemination stage, researchers worked with CHIVA to agree how best to communicate the results of the study. These results showed that, for young people whose HIV treatment includes the drug efavirenz, it may be safe in the short-term for some young people living with HIV to have a short break from HIV medicines at the weekend. But more evidence about the long-term safety of this strategy is needed before this approach is used outside of a clinical trial.

Because this message is complex, it was agreed to produce a handout explaining the results to give to children and young people who took part in the trial. Other options were considered (e.g. a film

targeted at all children and young people with HIV, not just those who took part in this study), but considered not to be appropriate at this stage.

CHIVA helped to prepare the handout for use in each country, particularly by working on the graphics and giving feedback on the text. The handout was translated and used in all of the countries where the trial took place, with the exception of Uganda, where a dissemination meeting was held for the children and young people who took part in the trial to tell them about the results.

The costs of PPI (both financial and time)

CHIVA was paid around £2,000 for their help with the dissemination stage of the study. This was felt to represent good value for money.

What impact did the involvement have?

The study meetings in Uganda were particularly successful. Children and young people who have taken part have said they enjoy these meetings. They felt that their contribution had been valued, and they valued the chance to meet with other HIV-affected children and young people. The study meeting also enabled researchers to introduce and endorse a new qualitative researcher.

At the dissemination stage the involvement of CHIVA was very useful. CHIVA has experience of communicating with children and young people and particularly in thinking about and designing graphics to do this. They were able to identify anything that was unclear on the draft handout about the results of BREATHER. They made the handout more accessible and more professional.

Plans for the future

Researchers at the MRC CTU at UCL and at London School of Hygiene and Tropical Medicine are working with CHIVA to develop the involvement of children and young people in the UK in HIV research, by setting up a group of HIV+ young people who can get meaningfully involved in a range of HIV studies from an early stage. This would also enable children and young people to develop their skills and to offer each other support.

“Involvement has to be meaningful, so it’s important to involve children and young people from the outset. This can be challenging, because often funding isn’t available at this early stage.” Lecturer in Medical Sociology**,** London School of Hygiene and Tropical Medicine

“I would definitely recommend working with groups like CHIVA if you are thinking about how to share results with children and young people. Make sure you allow plenty of time for this. I would recommend setting up a small group from the TSC or TMG to work with patient reps on material to disseminate to trial participants about results.” PENTA Trial Manager

Systematic review and meta-analysis collecting individual patient data (IPD) from trials comparing radiotherapy-based treatments in cervical cancer.

What was the aim of the involvement?

When work began on this project, trials results suggested that women with cervical cancer lived for longer if they received chemoradiotherapy. However there were some concerns, both from the clinical community and from patients, regarding long-term side effects potentially associated with this treatment. The researchers were keen to involve women who had experienced treatment for cervical cancer, in order to:

- Inform the discussion about the treatments involved and in particular how side effects might impact on women's day-to-day lives post treatment.
- Gain a better understanding of what women might consider to be acceptable in terms of side effects, assuming that the treatment really did improve survival.

Who was involved?

Five women with personal experience of cervical cancer. Three of them were recruited with the help of a PPI advisory group that had been set up to help with this project. Two were recruited via Macmillan Cancer Support's CancerVoices website

They chose to be called 'Patient Research Partners'.

Why did patients choose to get involved?

The Patient Research Partners were eager to use their experience of cancer to help with research, and to support a project that was addressing a question that they thought was important.

What activities did people undertake?

Patient Research Partners chose how they wanted to be involved in the review. This included:

- Helping to trace contact details for trial investigators
- Learning about data management and analysis
- Contributing to project newsletters
- Attending the Collaborators' meeting, at which the first results were presented
- Providing input into the lay summary for the Cochrane systematic review and a "Key Messages" document
- Taking part in discussions about potential research on chemoradiotherapy side effects

The researchers became involved in a related research project based on a Royal College of Radiologists audit of the effects of treatment for cervical cancer, including associated side effects. Three of the Patient Research Partners wrote a joint editorial with the researchers to describe these side effects from a patient perspective.

How were patients supported?

Only one of the five women knew about systematic reviews, so the researchers developed information to help them, and constantly improved and added to it from their comments and feedback.

The costs of PPI (both financial and time)

This project had specific funding for PPI - £1,500 per year for three years. Involving the Patient Research Partners took extra time and effort – e.g. the information pack took time to develop and produce, but including these activities in the grant application meant we had the time and resource to do it. Also, using the materials from this project as a template for future projects should mean that less time is needed in future.

What impact did the involvement have?

The researchers evaluated the experience from both the Patient Research Partners and researchers' perspectives.

The greatest impact on the researchers was that the involvement of the Patient Research Partners directly led the researchers to get involved in another research project with a greater focus on late side effects for treatment. It also motivated them to publish an editorial with the Patient Research Partners.

The Patient Research Partners all agreed that their main impact on the research was in adding a viewpoint that otherwise would not have been heard. They also felt that their involvement might help to bring about changes, e.g. that future trials might collect better data on late side effects.

We worried about getting it right – what we were going to be able to talk about and pitching things correctly, but we built up a relationship with the research partners that led to open and informative discussions. Senior research scientist

The presentations and design of the materials took great care to unravel the complex world of research acronyms and concepts and explain complex ideas simply but without dumbing down. That made me feel that we were equal partners in a really important piece of work….Attending the conference with senior practitioners from all over the world was a very memorable experience for me. Understanding their priorities and their perspective on cancer treatment was very revealing and made the work with patient partners all the more essential to help build a holistic picture of people living with cancer and their needs… It's a good feeling from a patient perspective to have contributed to a piece of work which recognises the after effects of treatment and survivorship issues."

Patient Research Partner

Further information

Vale C et al, Evaluation of patient involvement in a systematic review and meta-analysis of individual patient data in cervical cancer treatment. Systematic reviews (2012) 1:23
www.systematicreviewsjournal.com/content/pdf/2046-4053-1-23.pdf

DART: a clinical trial about the best ways to manage anti-HIV therapy in the public health access programmes being rolled-out in Africa and other resource-limited settings.

What was the aim of the involvement?

DART was the largest trial of its kind in Africa, and one of the first HIV trials to take place in more than one African country and in a number of different clinics. It took place at a time when there were very few antiretroviral drugs available in Africa. Because of these things, researchers knew that it was vitally important to work in partnership with HIV/AIDS support groups from the planning stage.

Who was involved?

Two community representatives were recruited to the trial steering committee (TSC). In Uganda, the representative was the president of the market vendors' association of a very large market in Kampala, Uganda. The market vendors were campaigning for treatment when the trial was being set up, and the HIV AIDS association in Uganda nominated her to sit on the TSC. In Zimbabwe the representative specialises in community engagement with projects connected to the Clinical Research Centre at the University of Zimbabwe in Harare.

How were people involved?

As well as sitting as members of the Trial Steering Committee, both community representatives interacted directly with DART trial participants. The Ugandan representative gave every participant in Kampala her phone number, and worked with participants to set up peer support groups in all of the centres where the trial was taking place.

In Zimbabwe, where there was one centre that recruited participants, peer support groups were also set up.

As well as offering support to participants, these groups enabled researchers to keep people up to date with the progress of the trial and any changes that were taking place, and at a later date to tell people about the results of the trial. The groups have continued to meet after the trial results were published.

The costs of PPI (both financial and time)

The representatives on the Trial Steering Committee were paid travel expenses – this was particularly important as the trial was based in more than one country. The representatives were not paid for their time.

The involvement was seen by researchers to be extremely cost effective.

What impact did the involvement have?

Community involvement in this trial was invaluable. It was a key contributing factor to the very high levels of recruitment and retention in the trial. But perhaps more importantly the involvement led to a sense of a DART community. This made it much easier to tell people about the results of the trial, and to do more research with and amongst these communities. For example, the ARROW trial was developed in response to the DART trial.

"From the perspective of doing research in Africa, community representatives are really important as they can help you to engage with patients and participants, and tell you about traditional structures. You need to involve community representatives at a really early stage, before you've chosen the trial sites." Trial Manager

“The choice of community representatives for trial steering committees is vital. You need someone with community links who is highly respected.” Professor in Epidemiology

Microbicides Development Programme: an efficacy and safety trial of a candidate vaginal microbicide to reduce the risk of HIV infection for women

What was the aim of the involvement?

The Microbicides Development Programme (MDP) was a partnership of European and African researchers set up to develop safe, effective, acceptable and affordable vaginal microbicides for the prevention of HIV acquisition. The MDP undertook feasibility and pilot studies in preparation for a clinical trial to test the effectiveness and safety of two different concentrations of a candidate microbicide in Africa. Social science research was integrated in the trial to assess the acceptability of vaginal microbicides to women, their partners and the wider community.

The trial needed to recruit almost 9,400 HIV negative women in order to achieve reliable results. The trial was conducted by six research partner centers in Africa - South Africa (3 centres), Tanzania, Uganda and Zambia.

The feasibility study was designed to measure if the centres could recruit women to take part in the trial, and maintain their involvement for a year or more. It also aimed to establish community structures to support the trial.

Who was involved?

From the outset, the MDP prioritised community engagement and collaborated with Southampton University to coordinate community liaison activities at each centre. At least one community liaison officer (CLO) was appointed at each of the trial centres with a dedicated CLO budget. Many of the research centres had a Community Advisory Board already in place. In some centres the Boards were encouraged to appoint more women in preparation for the microbicide trial. In two centres, where there were no pre-existing Community Advisory Boards (CABs) or groups, they were set up from scratch. The configuration of the community advisory boards differed across the centres depending on the local circumstances. In some the CAB consisted of community representatives elected by their residential committees or selected from each residential ward, in others CABs included Department of Health officials or media representatives. In others CABs were made up of study participants. One centre supplemented their locally elected institutional CAB with a study participant stakeholder group and six monthly meetings with local institutional stakeholders such as the Department of Health, Traditional Authority, and Municipality. The CLOs, in collaboration with the CAB members, used a range of community outreach strategies to involve a wide range of local stakeholders and community members.

How were people involved?

Each centre took a different approach to community and participant involvement in response to the local context. At all centres, involvement was extensive extending from the beginning of the feasibility study all the way through to the end of the clinical trial. Many centres adopted a participatory learning and action approach.

Throughout the study, CAB members and other stakeholders were involved with developing or reviewing study materials, procedures, community involvement strategies, and study policies such as standard of care, reimbursement, and results dissemination.

Each centre ‘mapped’ organisations in their areas that were working in the field of HIV, sexual health and reproductive health, as well as organisations or places where women who might take part in the study were working and/or living. They made links with relevant communities and community groups to talk with people about the study and to seek their views about it.

For example in Mwanza City, Tanzania, women working in food outlets and recreational facilities were at higher risk of HIV infection, so the team mapped these facilities and talked to the women who worked at them about the study. This informed the facilities (bars and guesthouses) at which the team established weekly study clinics. Community representatives were elected to represent

different areas and types of facilities, and they formed a Community Advisory Committee for the study. Workshops and community meetings were then held regularly (every 4-6 months) to listen to women's views about the study and to make changes in response to these views. A detailed report of the approach adopted in Mwanza is available here and here.

Centres also used a range of outreach activities to recruit to the study. Study teams went to places where women who might be interested to join the study might be – for example in Durban they went to taxi ranks and community meetings to talk about the study. The Africa Centre in South Africa, had monthly community roadshows, that used edutainment techniques to engage communities in a discussion about the research studies. The MDP CLO would present details of the study either by talking about the study or performing songs or plays about the study. At the end of the presentations they would engage community members in question and answer sessions and give away prizes such as study T-shirts. Anyone interested in joining the study was encouraged to visit one of the community based study clinics.

Many centres used particular strategies to engage men in a discussion about microbicides and the MDP study, for example by organising football tournaments and presentations at male dominated work environments such as sugar mills, taxi associations, police stations or army bases. Some centres also involved traditional leadership groups and traditional health care practitioners in discussion about the study.

Community involvement was not viewed merely as a recruitment tool, but rather as key to the running of the study and maintaining a relationship with the community. As such, each team invested a lot of effort into feeding back the results of the study to the study participants and community members. In many centres, participants helped study teams develop the materials to feed back the results (visually, orally, with songs or plays). Feedback was achieved via community meetings, participant events, text alerts to participants, and information via print, radio and TV media.

Throughout the trial, the Community Liaison Coordinator produced quarterly reports on community involvement activity at each centre, collated community feedback for discussion on the monthly trial management group calls, and monitored action in response to negative feedback. MDP funded three international community workshops bringing together community engagement staff from each centre, along with representatives from community organisations and some of the centre social scientists. Those involved were able to share their approaches and learn from each other. Some centres also linked the CLOs with the Global Campaign for Microbicides (an international advocacy organisation) community engagement network so as they could share experiences with CLOs across a range of microbicide trial networks.

The costs of PPI (both financial and time)

Costs for Community Liaison Officers across the six centres and the international coordinator, annual international workshops for the community liaison officers, as well as expenses for involvement work were built into the budget for the MDP project from the outset.

What impact did the involvement have?

Community engagement at an early stage meant that researchers were able to make changes to the protocol and respond to issues raised by women who were considering taking part in the study. For example, in Mwanza, Tanzania, at the feasibility stage, concerns were raised about blood specimens being sold for witchcraft purposes; that specula used for examinations might not be clean; that transport allowances were inadequate and that there were delays in obtaining test results. The project responded by inviting members of the Community Advisory Committee to visit the laboratory to see how specimens were used and held community meetings to explain this; demonstrated how specula were cleaned and again discussed this in community meetings

(disposable specula were introduced for the trial); negotiated and raised reimbursement levels; introduced rapid HIV testing and streamlined laboratory reporting procedures.

Ongoing community engagement throughout the trial meant that researchers were able to monitor and respond to emerging issues. For example, in the Africa Centre, South Africa, the research team worked with the CAB and participant stakeholder group to develop and deliver responses to community concerns that emerged in response to the closure of a microbicide study conducted in another part of the province and to the closure of one of the MDP study arms. The community involvement resulted in swift and appropriate response to community concerns, avoiding any undue impact on study recruitment and retention. A paper about this is available here.

Without the work of the Community Liaison Officers and the local people involved in the community advisory boards and councils, the study would not have recruited or retained women successfully.

Although the study did not show a benefit for the microbicides being tested, the teams were able to successful complete the largest HIV prevention trial ever conducted on time and in budget, and positive lasting community relationships were established. This is vital when working with established community based research partners to ensure communities remain supportive of future research even after trials do not show positive results.

The experience of researchers and community members who were involved in the MDP enabled them to contribute to a number of resources that aim to help researchers engage with community members, including:

- *Good participatory practice: Guidelines for biomedical HIV prevention trials* by the Joint United Nations Programme on HIV/AIDS (UNAIDS).
- A number of implementation tools associated with the Good Participatory Practice (GPP) guide, including the Community Mapping Tool, GPP Circle Diagram and GPP Listing, Scoring, Ranking
- Examples in the *Communications handbook for clinical trials*
- Examples in the *Stakeholder engagement toolkit for HIV prevention trials*

"There is no point re-inventing the wheel – if there are already community structures, build on them. You need to look at everyone who might be affected by the arrival of a large study in an area – so mapping both health services and where potential participants may be and the groupings or networks they are part of is essential. In the MDP study, we learned that community engagement is best placed within the social sciences umbrella, but also that there needs to be strong links with clinic staff." Professor of Clinical Epidemiology

PIVOT: A randomised trial to compare the use of a single type of anti-HIV medication to the standard triple drug therapy, by monitoring the effect on disease progression, drug resistance and death in HIV infected patients.

What was the aim of the involvement?
There were three main reasons for wanting to involve people living with HIV and the HIV community more broadly in this trial:

- PIVOT was designed to be a large, UK based trial looking at the long term treatment of people living with HIV. Because the trial aimed to follow what happened to participants for five years, it was important to have involvement from the HIV community, so that they could encourage people to remain involved.
- It was possible that if the results showed that using one drug instead of three was just as effective, there was potential for the NHS to save money. The researchers didn't want the trial to be focussed on this. Rather, they wanted it to focus on potential patient benefit and they wanted to get this message across to people living with HIV.
- The trial funder, the Health Technology Assessment Programme, has a commitment to patient and public involvement in the research it funds. The research team would have involved people affected by HIV even if the funder had not been actively supportive of this, but it was a useful incentive and meant that funding for involvement could be included in the budget with confidence that this would be seen as a legitimate expense.

Who was involved?
The Chief Investigator approached the UK-CAB, a network for community HIV treatment advocates. He gave two presentations about the PIVOT trial when it was at the design stage and asked UK-CAB members to nominate one member to sit on the Trial Steering Committee and one to sit on the Data Monitoring Committee.

The chair of the UK-CAB, was appointed to the Trial Steering Committee (read his perspective below) and another member was appointed to the Data Monitoring Committee.

This was the first time that a community member had been appointed to a DMC for an MRC CTU trial. The Chief Investigator felt it was important to involve a community member in this way – there was some history of this type of involvement in HIV trials run from the USA, where it was felt to be helpful.

How are people involved?
In addition to the involvement of community members on the TSC and DMC, the PIVOT team worked with an organisation called The African Eye Trust to ensure that people of African origin living with HIV were made aware of the trial. Trust members gave presentations about the trial at a number of hospitals and clinics and featured an article in their magazine.

Once the trial was complete, all participants were invited to hear about the results of the trial at a meeting which immediately followed the results meeting for investigators.

The costs of PPI (both financial and time)

The financial cost of the involvement was in the region of £15,000. It was felt to represent good value for money. It is hard to quantify the amount of time spent on involvement, but again this was felt to represent good value for the trial.

What impact has the involvement had so far?
At the design stage, the UK-CAB made useful suggestions about the draft protocol. These were incorporated into the final design.

The patient representative was a central member of the TSC. He showed up to all meetings and took the role seriously, responding to requests for comments or assistance promptly. He made important contributions to the protocol and to draft publications. He provided a link between the HIV community and the trial. Because he is active in the HIV community he was able to pick up on themes and issues that were discussed at conferences etc. that were relevant to the trial and feed them back to Chief Investigator and the TSC.

Similarly the involvement of the patient representative in the DMC was felt to helpful.

It is likely that the involvement of the African Eye Trust had an impact on recruitment, as over 20% of trial participants were of African origin – this was a higher proportion than for many other HIV studies.

"For more controversial trials, involvement is essential, especially for long-term trials where the messages can change and where you need to retain participants to ensure reliable results.

"It's important to choose the people you involve carefully. You need to look for an existing reputable body, like the UK-CAB, where you can seek people's views. Try to avoid any research being monopolised by one or two people who may have strong views." Chief Investigator, PIVOT trial

The perspective of a community representative about involvement in the PIVOT trial steering committee

The interviewee is a former chair of the UK-CAB, a network for community HIV treatment advocates. He was a member of the PIVOT trial steering committee.

Why did you agree to get involved in this project?
I was diagnosed quite late with HIV. I was given a whole range of different drugs and knew that the side effects could be really unpleasant. The idea of the PIVOT trial – offering people one therapy instead of the usual three – was very appealing to me. And at the time I was on a mono-therapy, because of the side effects I had been experiencing.

I was the chair of UK-CAB when we were approached about this trial. We already had good links with MRC CTU, so it seemed a good idea to say yes to their invitation to get involved.

What did you hope to achieve? And did you achieve it?
UK-CAB was keen to have a seat on the Trial Steering Committee as we wanted to ensure that the trial kept a focus on the patient experience throughout. And on a personal level I wanted to learn more about clinical trials.

I felt I achieved both of these goals.

How were you involved?
I was a member of the Trial Steering Committee.

Did you receive any support or training? If so, what was this and was it helpful?
For this trial, UK-CAB agreed that it would be important to nominate someone to join the trial steering committee who already had some knowledge of HIV treatments and experience of representing the views of people affected by HIV.

I wasn't offered any formal training, but other TSC members were very helpful if I asked questions.

I think it's helpful if researchers ask patient and community reps what training they would find helpful.

Do you feel your involvement had an impact – on the project, on the researchers and/or on you?
I'd like to think so. I helped researchers get the language right when they were advertising the trial.

There was a keen interest to get more information about how well the single drug penetrated into the brain, to see if this might have an impact on brain function, particularly cognitive function. Initially, there was discussion about getting lots of people - maybe 100 or more - involved in this sub-study, which involved a lumbar puncture. After I flagged up that this was a big ask, the number for the sub-study was scaled down to 30-40, a number I still thought might be optimistic, and in the event, only 16 (I think) patients actually had a lumbar puncture.

There was also a lot of interest in cognitive function per se, and base-line tests were done on all trial participants when they joined the trial. An analysis was then done to see how cognitive function in people living with HIV (PLWH) (whether on mono therapy or triple therapy) compared with the general population. The results were presented in draft form for comment at an investigator's meeting, with a rather sensationalistic headline - about 50% of all PLWH showed signs of cognitive impairment compared to a much lower figure in the general population. I (and others) questioned not only the language used in the headline, but also raised concerns about the comparison with the general population. When corrections were made for socio-economic and cultural factors, the difference between results for PLWH and the general population was much less significant - hardly any difference. This reflects the fact that the PLWH tend to come from disadvantaged sections of the community – e.g. immigrants whose first language may not be English; people with chaotic lifestyles (PLWH are more likely to have drink/drug problems); PLWH are more likely to be suffering from depression, which can negatively impact cognitive function test results. The paper was published with a much more balanced view and a much more neutral headline.

The feedback meeting to participants highlighted a key issue - that of language. It is essential to use a different presentation for the participants than that used for the clinicians and researchers. Participants are not so much interested in the minutiae of the science, or the trial design and statistics, but really want to know the impact of the trial on clinical practice. I would advocate involving patient reps much more fully in the preparation of feedback for participants (something that time did not permit in the PIVOT trial).

Personally I learned a lot about how trials are run, and I was very impressed by the amount of planning and thought that goes into every step of the trial.

Any advice to other researchers thinking about PPI?
You need PPI at an early stage – especially in preparing any information for patients. It is really important to feed back the results of research to participants – I was pleased that this happened in the PIVOT trial. I'd like to see written information being sent out to trial participants about the results of trials too.

Any advice to people who are asked to get involved in research?
I'd encourage them to do it. You need to have an interest in research and to recognise that it can be quite hard work. Going through protocols takes time and effort. I've got a scientific background - it isn't necessary, but it has helped me.

Don't be afraid to ask 'stupid' questions – people are very helpful.

The perspective of a community representative about involvement in the PIVOT Data Monitoring Committee

The interviewee is a member of the UK-CAB, a network for community HIV treatment advocates. He was a member of the Data Monitoring Committee for the PIVOT trial.

Why did you agree to get involved in this project?
I applied to join the data monitoring committee of the PIVOT trial because it wasn't a straightforward trial of a new drug – it was looking at a new way of treating people, and whether that was safe and cost-effective. I have done some qualitative research, and was interested in being involved in some quantitative research. And I wanted to see how a data monitoring committee worked.

The UK-CAB asked people to formally apply to join the DMC.

What did you hope to achieve? And did you achieve it?
I wanted to put forward the patient's perspective, and especially how someone who might take part in this trial would see things. I did manage that, but I was surprised by how cautious other DMC members were. For example, we looked at a report where something bad had happened to one person, and people were very concerned. I agreed it was bad, but argued that we didn't have enough evidence to say whether this was down to the treatment, and we needed to wait to see if other similar reports came in. We were all agreed about that, but I felt my attitude to risk was different. As patients we are perhaps more prepared to take risks if we think there is also a potential for benefit.

How were you involved?
I was a member of the Data Monitoring Committee. We met every six months over the three years that the trial was running.

Did you receive any support or training? If so, what was this and was it helpful?
I was offered some background information when I got involved. But I had made it clear in my application that I have a masters degree in research methods, so I had some understanding about clinical trials. I talked with a friend who had been involved in another data monitoring committee and looked at information about other relevant trials.

Do you feel your involvement had an impact – on the project, on the researchers and/or on you?
Yes. I think I gave other committee members confidence in their decisions – if the patient rep was comfortable about doing or not doing something, then that increased the confidence of other members that they could be confident about this too. This was particularly the case at the early stages when we were looking at the outcome measures for the trial.

And my mental arithmetic was quicker than theirs!

Any advice to other researchers thinking about PPI?
It's a good idea to involve patients in the data monitoring committees. For the vast majority of trials their involvement won't make a huge difference, but for some trials, especially those where the side effects are an issue, they can be very helpful.

If the side effects of the treatments being tested are particularly horrible, patients can help to weigh up the risks and benefits. For example in one of the PIVOT sub-studies there was discussion about randomising people to have a lumbar puncture. I argued against that, because lumbar punctures can be very painful. Instead I suggested that lumbar punctures should only be used as part of the treatment.

If side effects are annoying but not debilitating, the patient representative may advocate continuing the study, because they know that the results could be beneficial for other patients.

The system that UK-CAB uses to select people to get involved in research is good, as it requires you to complete an application form and it enables UK-CAB to select people who are confident and able to get involved effectively.

Any advice to people who are asked to get involved in research?
Make sure you read any background papers beforehand.

Be confident enough to offer your views. I found that when I commented on something in the reports we'd been sent, other committee members would often say that they had been planning to raise the same issue.

Involvement in a data monitoring committee is great if you like thinking through problems, and if you are comfortable thinking about percentages and averages.

The PROUD Study: A randomised controlled trial examining the impact on gay men of using Pre-Exposure Prophylaxis (PrEP)

What's the aim of the involvement?

Pre-exposure prophylaxis (PrEP) is the use of antiretrovirals prior to exposure to HIV to prevent infection. PrEP is intended for use by people who may be at frequent risk for HIV. This includes people in high-risk behaviour groups such gay or other men who have sex with men who have anal intercourse without a condom.

When researchers at MRC CTU began to think about whether to carry out a trial in this area, they knew they needed to actively involve people who might benefit from PrEP, in order to ensure that any research undertaken would be acceptable as well as effective. MRC CTU has a history of involving people affected by HIV in our trials, so to some extent this involvement was a normal way of working.

Who is involved?

What makes the PROUD study different is the range and extent of involvement. At a very early stage the researchers worked with voluntary organisations that support people affected by HIV – the Terrence Higgins Trust (THT), NAM and GMFA, as well as the UK-CAB. But they knew they would also need input from men who were HIV negative, as this study was primarily aimed at therm. So the Terrence Higgins Trust and NAM put them in touch with a range of lesbian, gay and transgender organisations, and two of these – the Lesbian and Gay Foundation (LGF) in Manchester and Yorkshire MESMAC agreed to get involved.

PROUD is also different to many other clinical trials in its approach to involvement, as it has set out to actively involve trial participants in the study, asking them to give feedback on the study and to comment on the design of the full-scale clinical trial that was initially planned at the beginning of the study (as the PROUD study was designed as a pilot). This is an approach used more in international trials than in the UK and has been formalised in the Good participatory practice guidelines for biomedical HIV prevention. Participants are recruited by clinicians in participating centres – they have emailed flyers to participants and/or handed them out in their clinics. Participants also access a participant database to enter diary data and questionnaires , so adverts for participant meetings are also placed on this site. During the study the team introduced a Participant Mailing List that study participants can sign up to and receive updates including adverts for participant meetings.

How are people involved?

People have been involved in a variety of ways:

A Community Engagement Group
Early teleconferences to plan the study involved a broad range of voluntary organisations, a number of which formed the Community Engagement Group (CEG) including THT, NAM, the Lesbian and Gay Foundation (LGF), MESMAC, GMFA, the National AIDS Trust and later NAZ, reflecting the regional distribution of clinics involved in PROUD.

Trial Management Group
Representatives of the CEG are invited to join the monthly TMGs. Although community representation on this group is not constant, CEG members are copied into the study reports and join the calls when there are salient issues to discuss.

Trial Steering Committee

Three Community representatives –representatives from NAM, LGF and UK-CAB are members of the Trial Steering Committee. One of these representatives is the joint chair, along with a clinician.

Independent Data Monitoring Committee
The IDMC includes three members, with community representation from i-Base.

Participant Involvement Group
Between November 2013 and February 2015 there have been five topics discussed during nine meetings, involving just under 100 participants representing 12 of the 13 clinics involved in PROUD. Meetings have either been facilitated by PROUD study team members, members of the CEG (including representatives from NAM and THT), or other community partners (such as CliniQ).

What activities do they undertake?

The **Community Engagement Group** advises the trial team on community engagement activities, including recruitment materials (the study leaflet was designed by GFMA), communication and media activities. The Group meets before the Trial Steering Committee, so that it can inform discussions that take place there. CEG members offer community perspectives on all study decision making boards (the TMG, TSC and IDMC).

Participants are asked to advise on a range of study related topics. The discussion topics to date have included 'Future Options for PROUD' (addressing low recruitment to the pilot study and next steps for PrEP), 'Future HIV prevention research priorities' (including clinical research priorities for Truvada and future trial design options), 'Study design and data collection tools for a larger PROUD trial' (discussing the potential trial protocol including the continued acceptability of the study design and PPI reimbursement), 'PROUD study results' (which included discussing the results under embargo and advising on the dissemination of the study results including the Conference presentation and media release), and 'WHO PrEP Implementation Guidelines' (advising on key implementation priorities). Reports from each of the meetings are posted on the PROUD website and participant details are anonymised.

Members of UK-CAB were asked to comment on the draft participant information sheet and draft protocol.

The costs of PPI (both financial and time)

The PROUD study only received limited funding for the pilot stage – and there was no budget for PPI. Members of the Community Engagement Group have given their time and their premises free of charge, which has made a huge difference. Researchers have been able to pay for refreshments at meetings but to date have not been able to cover travel expenses. However they have ensured that people can access teleconferences free of charge.

The team noted the need to build in funding for PPI in any future application and discussed acceptable reimbursement rates during one of the participant involvement meetings.

A small amount of time (10 – 20 minutes) is allocated at each team meeting to discuss PPI. Time has also been spent planning for and taking part in meetings with community members and participants who have been actively involved, and in submitting an ethics application for participant involvement. It's hard to be precise about how much time this has taken – probably about 70-80 hours to date (August 2015).

What impact has the involvement had so far?

The involvement of people working in voluntary organisations through the Community Engagement Group has brought a level of expertise to the trial. The individuals from these organisations understand the science and have extensive networks they can draw on. The involvement of

participants has enabled the researchers to hear the perspectives of the potential end users of PrEP and get their input on the research priorities and study conduct. Even though this trial is at a pilot stage, this involvement has already had a significant impact:

- HIV charities were able to put researchers in touch with other relevant organisations. Their introductions meant the researchers found it easier to build effective working relationships with organisations they had never previously worked with
- All of these organisations helped the researchers to design the PROUD study. In particular they agreed that PrEP needed to be evaluated in the UK as part of a clinical trial
- At the design stage, they gave researchers confidence that the trial was feasible
- Also at this early stage, they advised researchers that they thought it was acceptable to proceed with a deferred arm (some men who take part in this trial are not offered the drug for one year). This was crucial for the development of the trial. They agreed that there should not be a placebo arm.
- Originally there had been a proposal to offer event-driven PrEP as well as daily PrEP. Community organisations did not view the event driven option as a priority, and it was later dropped.
- UK-CAB members re-wrote the participant information sheet to make it more user-friendly. They also suggested that researchers change the eligibility criteria to allow men who are active partners in anal sex to take part in the trial, as well as those who are passive partners.
- GMFA designed a leaflet to promote the study. All CEG members also promoted the study broadly.
- In April 2013, the Trial Steering Committee expressed concern that the trial was recruiting too slowly. The Community Engagement Group held a meeting to discuss this and as a result the HIV charities released a joint statement outlining their position on PrEP. This resulted in a number of media articles about PrEP, as well as a series of awareness messages by THT on GRINDR. Recruitment has subsequently improved – this may not be just as a direct result of this initiative, but it certainly helped.
- Participants were also asked about slow recruitment at the first participant involvement meeting. Their view was that slow recruitment was mainly due to not enough men being aware of PrEP or PROUD, not a result of men not being interested in PrEP or the PROUD study. They advised that researchers should not rush to broader roll out without understanding more about adherence and the impact of PrEP on sexual behaviour. All participants agreed that PrEP should only be available in dedicated clinics (like in PROUD) and supported plans for a larger clinical trial. This endorsement was seen as key by the Trial Steering Committee in their decision to go forward to seek funding for a full trial.
- Participant input was invaluable in improving the study schedule, data collection tools, and reimbursement policy from the participant perspective. Their input strengthened the funding application, however this was subsequently withdrawn as the key question of PrEP effectiveness was unexpectedly answered during the pilot study.
- The study team were aware that the release of the study result in February 2015 could have a direct impact on public health policy in the UK. The experiences in the USA have demonstrated the sensitives around the discussion of new HIV prevention technologies, concerns about displacing the prioritization of condoms in HIV prevention, and the risk of stigmatising potential users. Input from study participants, CEG members, and the broader public health field was essential in terms of ensuring the messages were clear, simple and effective. It also ensured sufficient numbers of participants were fully aware of the results and their willingness to share their views with the media at the time of the release increased interest and attention on the release and resulted in a number of compelling stories.

- After the release, CEG members and study participants advised on the story line for a documentary about the results, contributed to the making of the film, and continued to participate as panel members in public viewings of the film.
- The most recent participant involvement meeting discussing the WHO PrEP implementation guidelines highlights the way in which PPI structures within the study can also serve as a conduit for early-adopters of new technologies to contribute to guidelines for future roll out of effective options.

"This involvement is critically important in prevention studies. You've got to understand and connect with your target audience who don't perceive themselves as 'patients', so you can't reach them through their doctors as you would in a treatment trial. If you can't connect with them directly at the design stage, get as close as you possibly can. And remember that clinicians aren't usually the participants!" Professor of Clinical Epidemiology

"Involve as many people as early as possible, and keep the involvement dynamic." Social Scientist

The perspective of a community representative about involvement in the PROUD study

The interviewee works for NAM, a voluntary organisation that produces information about HIV and AIDS.

Why did you agree to get involved in this study?

I was invited by the Chief Investigator. In 2011 she and I made a joint presentation about community involvement in HIV prevention trials at a conference, and she invited me to be involved.

What did you hope to achieve?

If you're doing research about the prevention of sexually transmitted infections, it's important to know your target population, how they can be reached and what will and won't work. There have been studies in the past where researchers had failed to communicate the aims of the study, and these studies had low adherence. We wanted to make sure this study worked.

Do you feel your involvement had an impact?

This study wouldn't have happened without community input. We influenced the design of the study and the establishment of a community engagement group. We also put out flyers and articles in the gay press. That has helped to generate interest and to promote recruitment.

Any advice to other researchers thinking about PPI?

Researchers need to be politically aware, especially if they are doing research in an area that is potentially contentious. You need to sound out who people are, who's likely to be pro and anti the research. If the research is about an intervention that could have an impact on a population, you need to connect with that population and not just with experts.

Involvement is not tokenism – whether your study stands or falls can depend on the involvement of community groups.

Any advice to people who are asked to get involved in research?

You may have to clue yourself up very rapidly about the science. You need to realise that the researchers are the experts in designing trials, you are the experts in how to promote it.

The level of involvement may vary - it doesn’t have to involve lots of time. It doesn’t matter if you don’t know about science, if you’re a participant you’ve got really useful information to offer.

The QUARTZ study: A randomised controlled trial examining whether, in lung cancer which has spread to the brain, not giving radiotherapy is acceptable in terms of survival and quality of life.

What was the aim of the involvement?

The researcher who took the lead on this trial for MRC CTU was keen to put together a Trial Management Group that was as inclusive as possible. He was also influenced by colleagues who had involved patients in research projects and found it to be helpful.

Who is involved?

The trial lead recruited someone with personal experience of the disease, (his father died of lung cancer) who was an active member of the National Cancer Research Institute Consumer Liaison Group and the North Trent Consumer Research Panel.

How are people involved?

The lay representative sits on the Trial Management Group, and has done so from a very early stage.

The costs of PPI (both financial and time)

It has taken very little additional staff time to involve the lay representative in the TMG. Whilst QUARTZ had a budget (funding ran out before the end of the trial because it was slow to recruit), the representative was paid at the MRC CTU rates for his time and travel expenses were also covered. QUARTZ has also paid for him to attend conferences, where relevant.

What impact has the involvement had so far?

The lay representative has been a central member of the TMG. At points in what has been a challenging trial he gave the researchers the confidence to keep going. He has given a lot of talks about the trial to a range of audiences - having a lay representative talk about the trial and say it's worth joining has also given confidence to prospective participants.

"Good PPI depends on the skills of all of the people who are involved in the project. Researchers should have enough experience and empathy to be able to see all of the issues for a potential participant but in practice even the best researchers are likely to benefit from having someone look at the project specifically from a patient's perspective. Participant representatives are able to say, if you approach me about this trial, this would be my reaction and that could make a major difference to recruitment." Senior Scientist

The perspective of a lay member about involvement in the QUARTZ study

The interviewee has chaired the National Cancer Research Institute Consumer Liaison Group and the North Trent Cancer Research Panel. He got involved in cancer research because both of his parents were cancer patients.

Why did you agree to get involved in this study?

QUARTZ was one of the first studies I got involved in when I started to get interested in research. The lead researcher invited me to join the TMG, and I asked him to send me some background information. It was very short – so I was in right at the very early stages of the trial. I think that's

fantastic practice, something that other people should learn from. I was assured that I would be an equal member of the Trial Management Group, and that has proved to be the case.

The study attracted me because it focussed on quality of life, and it looked at treatment options for people who are nearing the end of their lives. I felt there was genuine equipoise, as I'd heard such differing views about the treatment being offered I the study.

What did you hope to achieve?

I wanted to bring a patient perspective to the study – not just my perspective, but the views of people I was in contact with – e.g. members of the Consumer Research Panel I'm part of.

Do you feel your involvement had an impact?

The patient is there to ensure that the research remains focused, that it's answering a question that's important, and that it's answering it for the right group of patients. And to make sure that when the researchers and the clinicians are talking about issues around the trial, someone who's lived through the illness will be able to have an input and to keep that opinion at the forefront of the trial.

It's not always easy to measure the impact of involvement or to put your finger on changes that came about because of your input. But I have been a member of the TMG for 10 years and I feel my contribution has been respected and valued. At the early stages I was told that I had made a useful input to the protocol.

One part of the QUARTZ trial is that people are telephoned to ask about their quality of life. Based on my experience with my parents, I asked whether you would get the same answers about quality of life from the patient and from the carer. It was decided that QUARTZ would collect quality of life data from the patient and from the carer and compare the two. So that will show whether or not you can use the carer's perspective on quality of life as proxy for the patient.

I know that I have also had an impact on recruitment. I have been invited to speak at lots of different meetings about QUARTZ – clinicians' meetings, investigators' meetings and so on. One clinician came up to me after I gave a talk about QUARTZ and said his hospital would be signing up to recruit patients as a result of my talk.

Any advice to other researchers thinking about PPI?

Do it. But think carefully about who you will involve – look for people with strong links to a patient group. If you get it right, patient involvement offers tremendous value for money.

Any advice to people who are asked to get involved in research?

If you agree to get involved, you need to be willing to take part in training. It's not just about attending meetings once or twice a year.

SORCE: a multi-centre phase III double-blind placebo-controlled study designed to examine the efficacy and tolerability of sorafenib (Nexavar) in patients with resected primary renal cell carcinoma at high or intermediate risk of relapse

What's the aim of the involvement?

Researchers working on this study had had positive experiences of involving people affected by cancer in colorectal studies, including CR07 and COIN. This led them to think about PPI in this kidney cancer trial.

Who is involved?

The Chief Investigator for the trial recruited a patient representative to become involved. He was chosen because of his experience as a lay member of the National Cancer Research Institute's Renal Cancer Clinical Studies Group.

How are people involved?

The patient representative sits on the Trial Management Group, and has done so from an early stage in the trial. He commented on the patient information sheet and looks at all amendments, especially those regarding safety.

The Chief Investigator for the trial, who chairs the TMG, ensures that he is included in discussions. And because he has previous experience of membership of this type of group, he is able to ensure that his views are heard.

The costs of PPI (both financial and time)

It has taken very little additional staff time to involve the patient representative on the TMG. He is paid at the recommended MRC CTU rate for his time, and his travel expenses are covered for attendance at face-to-face meetings.

What impact has the involvement had so far?

It has been very important to get a patient's perspective, especially in the development of the patient information sheet. This has helped to ensure that the information given to people is as understandable as possible.

His presence on the TMG has made people think about how they discuss things. His involvement has reminded other TMG members why this trial is taking place. His involvement will also be key at the analysis stage, and particularly in writing up a summary of the results in plain English that can be shared with participants and others.

"Take time to explain things in person to the people you involve. Don't leave them on their own. Remember that some discussions will affect patients who get actively involved on a much more personal basis." Project Lead

The perspective of a patient representative about involvement in SORCE

The interviewee is a patient representative on the SORCE Trial Management Group and a trustee at Kidney Cancer UK.

Why did you agree to get involved in this study?

I was approached by the Chief Investigator. He wanted a patient representative to sit on the TMG. The chair of Kidney Cancer UK had recently died and I picked up on some of the things he had been doing. I was delighted to be involved in this trial, because at the time it was one of the few kidney cancer trials that were available in an adjuvant setting. I thought it was an extremely positive and well-thought out trial.

What did you hope to achieve?

I wanted to represent the views of patients. And I wanted to get a handle on how patients were dealing with this new class of drugs. I had had kidney cancer and was classified as being at risk of relapse, so this trial was of interest to me on a personal level too.

It was a steep learning curve for me. I attended a couple of workshops (including one at MRC CTU). The terminology was challenging at the beginning and the teleconferences are fast, so it was hard to keep up at first. But you pick things up as you go along.

Do you feel your involvement had an impact?

I didn't have any influence on the trial design as it was already very well thought through when I went to my first meeting.

The major difference between the patient rep and other members of the TMG is that other people are tied up in the technical details of the trial. That means they can lose sight of the fact that patients need to agree to join the trial in order for it to happen. My role is to ask, if I was a patient, would I join this trial? If I was on it, would I continue to be on it?

I wouldn't exaggerate my contribution. I commented on different versions of the patient information sheet as the trial progressed. The drafts were usually very good, but I advised trying to avoid information overload, and I picked up things that I thought would be hard for patients to understand.

Sorafenib is very toxic so I have taken an interest in what can be done to try to prevent side effects. I have also been involved in thinking about ways to encourage patients to stay in the trial.

I hope to be involved at the results stage. These drugs are very expensive and if they work it will be important for them to be approved by NICE.

I have been involved at the early stages of a follow-up study (called RAMPART), through membership of the trial development group. This is a multi-arm trial and I am pushing for inclusion of a particular arm that I think patients would value most.

Any advice to other researchers thinking about PPI?

Adopt the same approach as the people who run the SORCE trial – I've been impressed by the way I've been included in this and other trials.

Allow time for patient reps to tune in at the beginning of their involvement. It might be useful to offer some statistics training at the analysis stage. It would also be helpful to visit a hospital to see how a trial works on the ground – that would bring the practicalities of running a trial to life.

Any advice to people who are asked to get involved in research?

I wouldn't exaggerate the contribution patients can make to trials, as lots of what is involved is very technical. But don't be inhibited by the technical side of things. There are good reasons to get involved - representing patients' views is important. Don't expect to become highly technically skilled – that's not your role.

STAMPEDE: A randomised controlled trial to find the best treatment for men with advanced prostate cancer

What's the aim of the involvement?

STAMPEDE is a large, complex RCT with a number of different treatment arms. Researchers working on this trial wanted patients to contribute to a number of different aspects of this trial.

Who is involved?

Planning for STAMPEDE began in 2002, and a patient representative was involved from the very first discussion meeting. Sadly, the first patient representative died before the trial was up and running. Two new patient representatives were recruited in his place. They both had some knowledge of trials and clinical research, as they sat as lay members on the National Cancer Research Institute (NCRI) Prostate Cancer Clinical Studies Group. They were also actively involved in prostate cancer patient groups.

They sat on the Trial Management Group for around five years, but then one died and the other retired. They were replaced by two new patient representatives, who were recruited via an advert distributed by Prostate Cancer UK.

How are people involved?

Two patient representatives sit on the Trial Management Group, and have been involved at all stages of the trial, from its inception. They have helped to:

- think about the questions the trial would address, the outcome measures and how the trial might work in practice
- plan and promote recruitment
- consider how best to explain what the trial is about
- shape and checked patient information sheets

They have also acted as co-authors on a number of papers.

The costs of PPI (both financial and time)

It is hard to quantify the amount of additional time it has taken to involve patients in this trial, but it has not been hugely time-consuming. At the beginning of someone's involvement it can take more time, as there needs to be explanation about the trial and the role of the TMG, and but as their involvement continues it takes very little additional time to involve them.

Patient representatives are paid at the recommended MRC CTU rate for their time, and their travel expenses are covered for attendance at face-to-face meetings.

What impact has the involvement had so far?

Just before the trial launched, there were problems with one of the trial drugs; STAMPEDE was investigating a number of drugs including one called celecoxib, from a family of drugs called cox-2 inhibitors. Just before the trial started to recruit, a drug in the same family was suddenly withdrawn from the market. All trials involving this family of drugs were stopped globally and indefinitely. The TMG had to decide whether to drop this drug and continue with the remaining arms, or to wait until there was further. The two patient representatives were key in the decision to wait so that the drug could be assessed, and in assessing the relative risk of the drug.

They also wrote articles in patient support group newsletters to help to promote the trial, and provided invaluable input to the development of patient information.

“Having two patient representatives on the trial management group is good – they can support each other. This is especially helpful in trials which are looking at treatments for conditions that are life threatening, as you may need to involve a series of people through the lifespan of the trial.

“We have learned that in the context of written information for patients, the role of our patient representatives is to point out what isn’t clear, not necessarily to make this information clearer.” Senior Scientist

“It can help if patient representatives have some kind of scientific background – being from a medical background is not necessary but having a scientific problem-solving mind set can be extremely beneficial.” Trial manager

The perspective of a patient representative about involvement in STAMPEDE

The interviewee is one of two patient representatives on the STAMPEDE Trial Management Group.

Why did you agree to get involved in this study?

I received an email from Prostate Cancer UK advertising this opportunity. From my own experience of prostate cancer and how things were explained to me, I knew it was pivotal to have a patient perspective. I am a researcher, so involvement in a research study appealed to me.

When I first got involved I had a meeting with the trial manager and the other patient representative. That broke the ice. It would have been helpful to have had a STAMPEDE specific glossary as some of the terminology can be challenging.

What did you hope to achieve?

From a personal perspective, I wanted to understand more about the pros and cons of clinical trials. I also wanted to contribute a patient perspective, especially to the patient information sheets and consent forms.

Do you feel your involvement had an impact?

Yes. I feel I have influenced the language used on the patient information sheets.

Any advice to other researchers thinking about PPI?

If you’re starting from scratch, think about what you want people to do. For example, how much do you want them to contribute to the design of the trial? The most successful research is research that is done ***with*** people because they will point out the elephant in the room.

Any advice to people who are asked to get involved in research?

Go for it. You need curiosity more than anything else – I have a background in maths and physics but that was a long time ago.
